# Non-syndromic Developmental Facial Palsy Co-occurring With Chiari I Malformation: Parallel Manifestations of a Shared Prenatal Disturbance?

**DOI:** 10.7759/cureus.108906

**Published:** 2026-05-15

**Authors:** Adrian A Naoun, Yandy Garcia Martin, Ernesto Garcia Santiago

**Affiliations:** 1 Medicine, San Juan Bautista School of Medicine, Caguas, USA; 2 Medicine, Hospital Menonita, Caguas, USA

**Keywords:** chiari i malformation, cranial nerve vii palsy, embryonic vascular disruption, facial palsy, posterior fossa

## Abstract

Congenital facial palsy most commonly results from perinatal trauma. Developmental causes are rare and typically occur within syndromic constellations such as Möbius, Goldenhar, or CHARGE (coloboma, heart defects, atresia choanae, retarded growth, genital abnormalities, ear anomalies). Non-syndromic developmental facial palsy (DFP) is exceedingly uncommon and is thought to arise from disturbances in facial motor nucleus development, the intrapontine facial nerve tract, or its vascular supply. Chiari I malformation (CM-I) is characterized by ≥5 mm cerebellar tonsillar descent below the foramen magnum, producing posterior fossa crowding and impaired cerebrospinal fluid dynamics. CM-I-associated cranial neuropathies, including facial nerve involvement, have been reported in the setting of secondary structural pathology such as syringomyelia, syringobulbia, or basilar invagination. We report a 37-year-old woman with a lifelong House-Brackmann grade III left facial palsy, documented at first cry and stable since birth, who presented with a six-month history of Valsalva-exacerbated occipital headaches, positional vertigo, and posterior cervical allodynia. Magnetic resonance imaging demonstrated 12-mm tonsillar ectopia with brainstem compression, without syrinx, syringobulbia, or basilar invagination, and incidentally revealed a thin elevated corpus callosum with mild ventricular prominence. Posterior fossa decompression with suboccipital craniectomy, C1 laminectomy, and autologous duraplasty resolved her CM-I symptoms. After an extensive literature review, we found no prior reports of non-syndromic DFP coexisting with CM-I in the absence of syringomyelia, syringobulbia, basilar invagination, or syndromic features. Although coincidence cannot be excluded, the corpus callosum and ventricular findings suggest a shared prenatal disturbance. These features may reflect parallel manifestations affecting overlapping developmental programs during ontogeny, rather than a direct causal relationship. A systematic framework comprising neuroimaging, electrodiagnostics, and targeted genomic profiling in similar cases may elucidate a developmental pattern from isolated coincidence, with potential implications for early surveillance.

## Introduction

Congenital facial palsy is the most common neonatal cranial neuropathy, with a reported incidence of approximately 0.8 to 2.1 per 1,000 live births [1,2]. Most cases are acquired and result from perinatal trauma, particularly forceps-assisted delivery [1]. A smaller subset, estimated at 8% to 14% of pediatric facial paralysis cases, arises from developmental mechanisms and is present at delivery [3]. Developmental facial palsy (DFP) most frequently occurs within syndromic constellations such as Möbius, Goldenhar, CHARGE (coloboma, heart defects, atresia choanae, retarded growth, genital abnormalities, ear anomalies), and hemifacial microsomia [4,5]. In contrast, non-syndromic DFP refers to isolated congenital facial weakness without an identifiable associated disorder [6,7]. The proposed mechanisms include abnormalities of the facial motor nucleus, the intrapontine facial nerve tract, or their vascular supply [7,8].

The facial motor nucleus and associated fascicles derive from the basal plate of the rhombencephalic neuroepithelium [9]. In contrast, the surrounding osseous and meningeal structures, including the cranial base, originate from paraxial mesoderm-derived occipital sclerotomes [10,11]. Arterial supply to the caudal pons and facial motor nucleus arises from pontine perforators of the basilar artery and branches of the anterior inferior cerebellar artery [12,13]. Definitive vascular architecture is established following the fusion of the primitive longitudinal neural arteries into the basilar trunk during weeks five to seven of gestation [12,13]. Disruption of these coordinated neuroepithelial, mesenchymal, and vascular developmental pathways may result in non-syndromic DFP, although the precise mechanism remains incompletely defined in individual cases.

Chiari I malformation (CM-I) is characterized by caudal descent of the cerebellar tonsils ≥5 mm below the foramen magnum on midsagittal imaging [14-16]. This displacement leads to posterior fossa crowding, impaired cerebrospinal fluid (CSF) flow at the craniocervical junction, and, in some cases, brainstem compression [16]. The leading pathogenetic model attributes CM-I to underdevelopment of paraxial mesoderm-derived occipital sclerotomes, resulting in a hypoplastic posterior fossa that cannot accommodate a normally developed hindbrain [10,11]. Emerging genetic evidence further implicates rare coding variants in chromodomain and paraxial mesoderm developmental genes as contributors to CM-I pathogenesis [17].

Morphometric studies support the hypoplasia hypothesis, demonstrating reduced posterior fossa volume and smaller foramen magnum dimensions in CM-I patients compared to controls [18]. Clinically, most symptomatic patients present with Valsalva-induced occipital headaches, neck pain, or myelopathy related to syringomyelia. Cranial neuropathies have also been reported, most commonly involving the abducens nerve and less frequently the facial nerve [19,20]. Notably, previously reported cases of CM-I-associated facial nerve involvement typically occur in the setting of syringobulbia or basilar invagination [20]. Cases without these secondary features remain distinctly uncommon. After an extensive literature review, we found no reports documenting the coexistence of non-syndromic DFP and CM-I.

## Case presentation

A 37-year-old Hispanic woman presented with a six-month history of progressive occipital headaches, positional vertigo, and posterior cervical allodynia. Her past medical history was notable for a left-sided lower motor neuron facial palsy present from birth, and a childhood seizure disorder (onset two to 10 years) in sustained remission. There was no family history of facial palsy, Chiari malformation, consanguinity, or recognized neurodevelopmental syndromes. The patient was born following an uncomplicated pregnancy (G1P1A0; maternal age 35 at delivery). Prenatal screenings, including routine obstetric ultrasounds, were unremarkable. There was no history of maternal infection, teratogen exposure, substance use, or known pregnancy complications. Delivery was spontaneous vaginal without instrumentation, forceps, vacuum assistance, or obstetric trauma. Left-sided facial asymmetry was identified by the delivery team and the patient's mother immediately following birth, first observed during the patient's initial cry, manifesting as deviation of the oral commissure and incomplete left-sided facial excursion. There was no preceding febrile illness, viral prodrome, otitis, herpetic eruption, or other infectious trigger in the neonatal period. The palsy remained stable in severity and distribution throughout childhood and adulthood, without progression, fluctuation, or episodes of partial resolution.

A seizure disorder emerged between ages two and 10 years, characterized by infrequent generalized tonic-clonic episodes occurring approximately once or twice per year. Baseline and subsequent electroencephalograms were reported as normal, without epileptiform discharges, focal slowing, or generalized spike-wave activity. Prior brain computed tomography and magnetic resonance imaging, performed during initial epilepsy workup in childhood, were reported as unremarkable apart from subtle ventriculomegaly and thinning of the corpus callosum. The patient received antiepileptic therapy for an unspecified period and achieved sustained seizure remission; antiepileptic drugs were discontinued decades prior to this presentation without any recurrence. The current presenting symptoms began approximately six months prior to evaluation. She reported occipital and posterior cervical headaches occurring three to five times per week, of throbbing and pressure-like quality, rated seven out of 10 in severity, and lasting one to four hours per episode. Headaches were consistently precipitated by Valsalva maneuvers, coughing, sneezing, straining, and postural changes, and were accompanied by nausea and positional vertigo. Photophobia and phonophobia were absent. She additionally reported intermittent posterior cervical allodynia to light touch.

**Figure 1:**
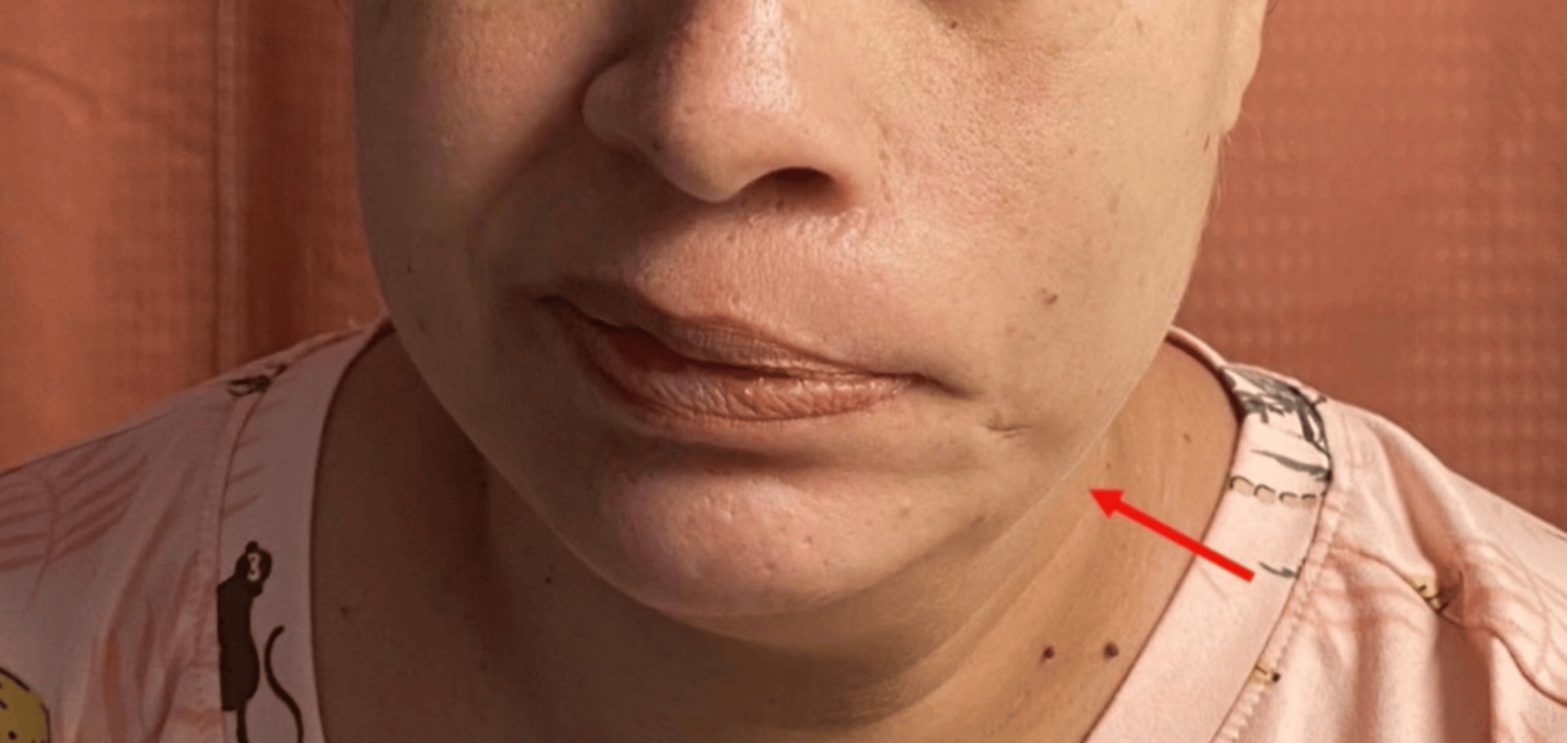
Frontal photograph of left House-Brackmann grade III facial palsy. Smile reveals left oral commissure deviation with blunted excursion and effaced nasolabial fold, consistent with moderate lower motor neuron weakness. Grade III = moderate, non-disfiguring weakness vs. II (slight) and IV (severe with synergistic closure).

On examination, the patient was alert and fully oriented. Neurological examination demonstrated a stable left-sided lower motor neuron facial weakness, graded as House-Brackmann III. Documented features included mild lagophthalmos on attempted forced eye closure, effaced left nasolabial fold, and blunted excursion of the left oral commissure on smiling (Figure 1). Consistent with House-Brackmann grade III, there was moderate but not complete weakness, with voluntary movement preserved across all three facial zones (frontal, ocular, and oral), preserved forehead wrinkling on the affected side, symmetric corneal reflex, and absent synkinesis or mass movement. Taste was intact over the anterior two-thirds of the tongue bilaterally, tearing was preserved, and there was no hyperacusis. The remainder of the cranial nerve examination was unremarkable. Motor, sensory, coordination, and gait examinations were normal, with the exception of mild sway on tandem gait. There was no dysarthria, dysphagia, upper motor neuron sign, or Parkinsonism. Electrodiagnostic evaluation, including nerve conduction studies, needle electromyography of facial musculature, and blink reflex testing, was discussed with the patient; however, she declined further workup given the lifelong stability of her palsy and its unambiguous presence since birth.

**Figure 2:**
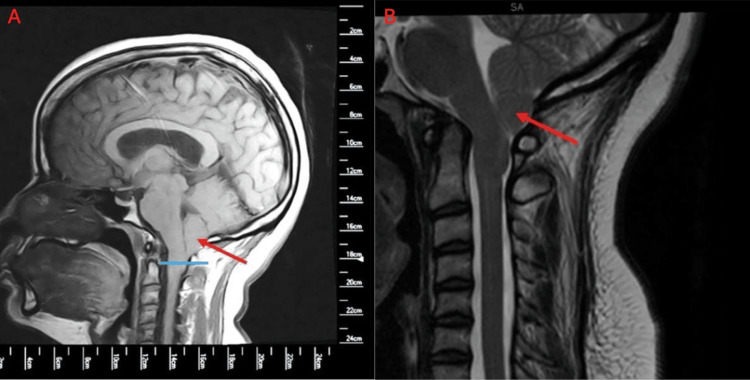
Pre-operative sagittal T1-weighted MRI A. 12-mm cerebellar tonsillar ectopia below McRae line (red arrow, blue line). B. Craniocervical junction crowding with obliterated cerebrospinal (CSF) spaces (red line)

Magnetic resonance imaging of the brain demonstrated 12 mm cerebellar tonsillar descent below the McRae line on midsagittal T1-weighted sequences, with obliteration of the cisterna magna and crowding at the craniocervical junction (Figure 2). There was no evidence of syringomyelia, syringobulbia, basilar invagination, hydrocephalus, or structural abnormality of the facial nerve, internal auditory canal, or temporal bone. The corpus callosum was noted to be thin and elevated, and the lateral ventricles showed mild prominence without obstruction, consistent with findings documented on prior pediatric imaging. Phase-contrast cerebrospinal fluid flow imaging was not performed.

The patient subsequently underwent standard posterior fossa decompression for symptomatic CM-I, consisting of suboccipital craniectomy, C1 laminectomy, and autologous duraplasty using a pericranial graft. No tonsillar coagulation, subpial resection, or intradural arachnoid dissection was performed. The procedure was uncomplicated, without cerebrospinal fluid leak, wound infection, aseptic meningitis, or bulbar dysfunction. Postoperative care included serial neurological examinations every four hours, midline head positioning with a soft cervical collar for 48 hours, avoidance of Valsalva maneuvers, and incentive spirometry. 

**Figure 3:**
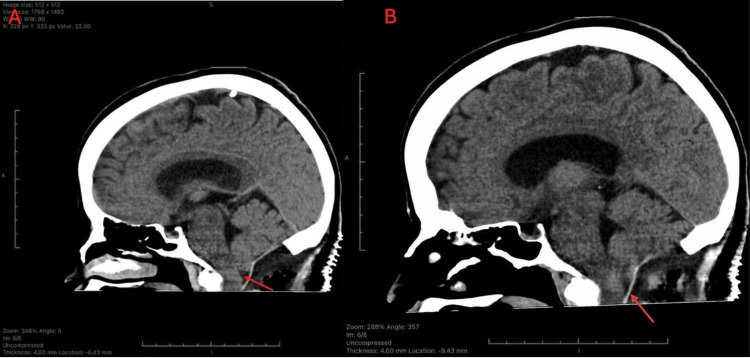
Post-operative sagittal head computed tomography (CT) following posterior fossa decompression and duraplasty. A. Demonstrates suboccipital craniectomy with bony decompression and expanded posterior fossa volume. The red arrow indicates the craniocervical junction with restored cerebrospinal fluid (CSF) spaces. B. Magnified sagittal view consistent with successful bony decompression and duraplasty without evidence of complication.

Head computed tomography on postoperative day 2 confirmed successful bony decompression, with adequate expansion of the cisterna magna, restored subarachnoid CSF spaces at the craniocervical junction, and no evidence of hematoma, pneumocephalus, hydrocephalus, or subdural collection (Figure 3). Consistent with the surgical technique performed, the posterior fossa decompression showed an enlargement of the available posterior fossa volume, thus facilitating dynamic restoration of the cerebrospinal fluid (CSF) around the tonsils. At three-month follow-up, the patient reported complete resolution of Valsalva-induced occipital headaches, vertigo, and posterior cervical allodynia. The left facial palsy remained unchanged at House-Brackmann grade III, consistent with a long-standing developmental lesion rather than an acquired compressive process reversible with decompression. The patient remained seizure-free off antiepileptic medication. There were no new neurological deficits, wound complications, or delayed CSF dynamic alterations. The patient reported substantial improvement in quality of life following surgery, particularly resolution of activity-limiting headaches and cervical pain.

## Discussion

CM-I shows variable penetrance and expressivity due to a multifactorial etiology, including genetic factors [17], posterior fossa hypoplasia from para-axial mesoderm underdevelopment [10,11], and environmental teratogen exposure [16]. CM-I exists on a morphometric continuum of small posterior fossa syndrome, from congenital crowding with minimal tonsillar descent to frank herniation [16,18]. Morphometric analysis of 78 CM-I patients and 75 controls showed that posterior fossa volume and foramen magnum diameter were the only parameters significantly reduced in CM-I [18]. These findings narrow the anatomically meaningful substrate of CM-I to a constrained osseous compartment [18]. Hindbrain compression effects manifest as cranial nerve dysfunctions, including Chiari-associated abducens palsy, facial weakness, hemifacial spasm, trigeminal neuralgia, and lower cranial nerve syndromes such as spinal accessory neuralgia [19-24]. Against this background, we report the case of a 37-year-old woman presenting with symptomatic CM-I co-occurring with a non-syndromic developmental facial palsy documented from birth. After an extensive literature review, we found that this combination has not been previously reported in the absence of syringomyelia, syringobulbia, basilar invagination, or recognized syndromic features.

To contextualize this co-occurrence, we first review the spectrum of CM-I-associated cranial neuropathies. Cranial neuropathies occur in 15%-25% of symptomatic CM-I patients, typically involving nerves IX-XII due to brainstem traction or compression [16]. Facial nerve involvement is comparatively uncommon and has been reported in several distinct mechanistic patterns. Pilon et al. described a 30-year-old woman with concomitant bilateral abducens palsies and bilateral facial weakness in the setting of CM-I with cervical syringohydromyelia, with full symptom resolution following posterior fossa decompression [19]. Massey et al. reported acute combined sixth and seventh nerve palsies in a four-year-old with CM-I, holocord syringomyelia, and pontomedullary syringobulbia [21]. Sherlock et al. presented the first pediatric case of unilateral CN VII palsy in a seven-year-old with CM-I and syringobulbia, similarly resolving after decompression [20]. Hemifacial spasm, an irritative rather than paralytic phenomenon, has been described in CM-I both with and without syringomyelia [22,23]. Trigeminal neuralgia has likewise been reported as a presenting symptom of CM-I, attributed to neurovascular conflict at the trigeminal root entry zone in the setting of posterior fossa crowding [24]. These reports establish that diverse cranial nerve syndromes can manifest in CM-I, unified by secondary structural abnormality mediating dysfunction through brainstem distortion [19-21]. Compellingly, our patient demonstrated none of these secondary features.

A separate central mechanism also warrants exclusion: Zhao and Ren described an "uncrossed central facial paralysis" from pontine perforator infarction, producing a forehead-sparing corticobulbar pattern inconsistent with our patient's tri-zonal weakness and partial frontalis preservation [25]. The temporal relationship between palsy and CM-I symptomatology offers a further distinguishing framework. The Pilon, Massey, and Sherlock cases share an acute or subacute palsy onset alongside CM-I symptoms with full resolution after decompression, thus consistent with acquired compressive or syringobulbia-mediated mechanisms [19-21]. The Decraene et al. series of six newborns with developmental unilateral facial palsy provides the inverse: facial nerve aplasia or hypoplasia on MRI in five of six cases, none with CM-I, framing non-syndromic developmental facial palsy as a primary nerve disorder rather than a posterior fossa phenomenon [26].

Granting a developmental etiology, two interpretations of the co-occurrence with CM-I merit consideration. The first is coincidence: CM-I has an estimated adult prevalence of 0.5% to 3.5% on MRI series [27,28] and non-syndromic developmental facial palsy occurs in fewer than one in 10,000 live births [7]. The second, more parsimonious interpretation is that both conditions reflect a shared prenatal disturbance affecting overlapping developmental programs of the posterior fossa and adjacent neural structures. Our patient's additional findings of a thin elevated corpus callosum and mild ventricular prominence support this framing by suggesting broader neurodevelopmental perturbation [29-31]. The corpus callosum forms between gestational weeks eight and 20, a window overlapping with hindbrain segmentation, occipital somite condensation, and rhombomere-derived cranial motor nucleus differentiation [9,29,30]. Concurrent disturbances across midline commissural development, posterior fossa mesodermal patterning, and rhombencephalic motor nucleus formation could plausibly arise from a single insult during this window without manifesting as a recognized syndrome. Under this framing, the developmental facial palsy and CM-I represent parallel rather than directly causal manifestations of an unspecified prenatal disturbance.

Several limitations warrant consideration. The patient declined nerve conduction, needle electromyography, and blink reflex testing, given the lifelong stability of her palsy. Renault demonstrated in 172 children with congenital facial palsy that facial EMG reliably distinguishes developmental from traumatic etiologies and characterizes lesion chronicity, axonal versus demyelinating nature, and distribution [27]. High-resolution facial nerve imaging (e.g., three-dimensional constructive interference in steady state) was not performed; targeted genetic testing was deferred based on patient preference and absence of syndromic features. As a single case, this report cannot establish causality. Rather, the proposed shared developmental window requires corroboration through systematic neuroimaging and genetic evaluation of similar cases.

## Conclusions

We report a 37-year-old woman with symptomatic CM-I and a non-syndromic developmental facial palsy documented from birth in the absence of syringomyelia, syringobulbia, basilar invagination, or syndromic features. Posterior fossa decompression resolved her CM-I symptoms without altering the facial palsy, consistent with the temporal dissociation between the two conditions. While coincidence cannot be excluded, the additional findings of a thin elevated corpus callosum and mild ventricular prominence raise the possibility that both entities reflect parallel manifestations of a shared prenatal disturbance affecting overlapping developmental programs during early gestation. Multimodal profiling comprising neuroimaging, electrodiagnostics, and targeted genomic profiling in similar cases will elucidate a developmental pattern from isolated coincidence, with potential implications for early surveillance.
